# Transpericardial Left Atrial/Left Ventricular Vent Placement During Robotic Mitral Surgery

**DOI:** 10.1093/icvts/ivag162

**Published:** 2026-05-29

**Authors:** Gokhan Arslanhan, Zeynep Sıla Ozcan, Murat Bastopcu, Anıl Karaagac, Halim Ulugol, Muharrem Kocyigit, Sahin Senay, Cem Alhan

**Affiliations:** Department of Cardiovascular Surgery, Acibadem Mehmet Ali Aydınlar University School of Medicine, Istanbul, 34638, Turkey; Department of Cardiovascular Surgery, Acibadem Mehmet Ali Aydınlar University School of Medicine, Istanbul, 34638, Turkey; Department of Cardiovascular Surgery, Acibadem Maslak Hospital, Istanbul, 34457, Turkey; Department of Cardiovascular Surgery, Acibadem Maslak Hospital, Istanbul, 34457, Turkey; Department of Anesthesiology, Acibadem Altunizade Hospital, Istanbul, 34662, Turkey; Department of Anesthesiology, Acibadem Mehmet Ali Aydınlar University School of Medicine, Istanbul, 34638, Turkey; Department of Cardiovascular Surgery, Acibadem Mehmet Ali Aydınlar University School of Medicine, Istanbul, 34638, Turkey; Department of Cardiovascular Surgery, Acibadem Mehmet Ali Aydınlar University School of Medicine, Istanbul, 34638, Turkey

**Keywords:** robotic mitral surgery, left ventricular venting, cardiac distension

## Abstract

Robotic mitral surgery is one of the most common robotic cardiac procedures being performed, which presents many benefits including smaller incisions, less postoperative pain, quicker recovery, decreased blood transfusion requirements for the patient, better exposure and better visualization of the mitral valve, and increased operative dexterity for the surgeon. As redo robotic mitral operations have increased in number, the use of atrial and ventricular sumps have also increased, especially during cases done under fibrillation. Our practical and easy-to-implement technique that consists of transpericardial atrial and ventricular vent placement during robotic mitral procedures may prevent dislodgement of the vents and may be beneficial to avoid important complications such as air embolisms and left ventricular distension resulting in subendocardial ischaemia.

Robotic mitral surgery is one of the most common robotic cardiac procedures being performed, which presents many benefits including smaller incisions, less postoperative pain, quicker recovery, decreased blood transfusion requirements for the patient, better exposure and better visualization of the mitral valve, and increased operative dexterity for the surgeon.^1^ However, there are some crucial points to consider during this procedure to avoid complications. Complete de-airing should be ensured and left ventricular distension should be avoided to prevent air embolisms and subendocardial ischaemia.^2^ Especially in mitral valve procedures done under fibrillation, the placement of an extra venting sucker across the mitral valve to prevent left ventricular distention, which is also left in place during rewarming to facilitate de-airing, is beneficial. Avoiding dislodgement of the vent during the procedure is crucial to ensure ventricular deairing is performed appropriately.

The classical set-up for the robotic mitral operations in our centre was as described previously.^3^ A mini thoracotomy is performed after the anaesthetic preparation is completed, usually at the right fourth intercostal space and of 4 cm length. Left and right working ports are inserted through the third and fifth intercostal spaces, respectively. A stab incision is made at the second intercostal space through the anterior axillary line, and the suction vents and a Chitwood clamp are placed through this incision (**Figure 1**). The port implantation is followed by the placement of a soft tissue retractor. The robotic system is docked after the trocars are positioned. The camera is sent through the soft tissue retractor, and cardiopulmonary bypass is started.

**Figure 1.**
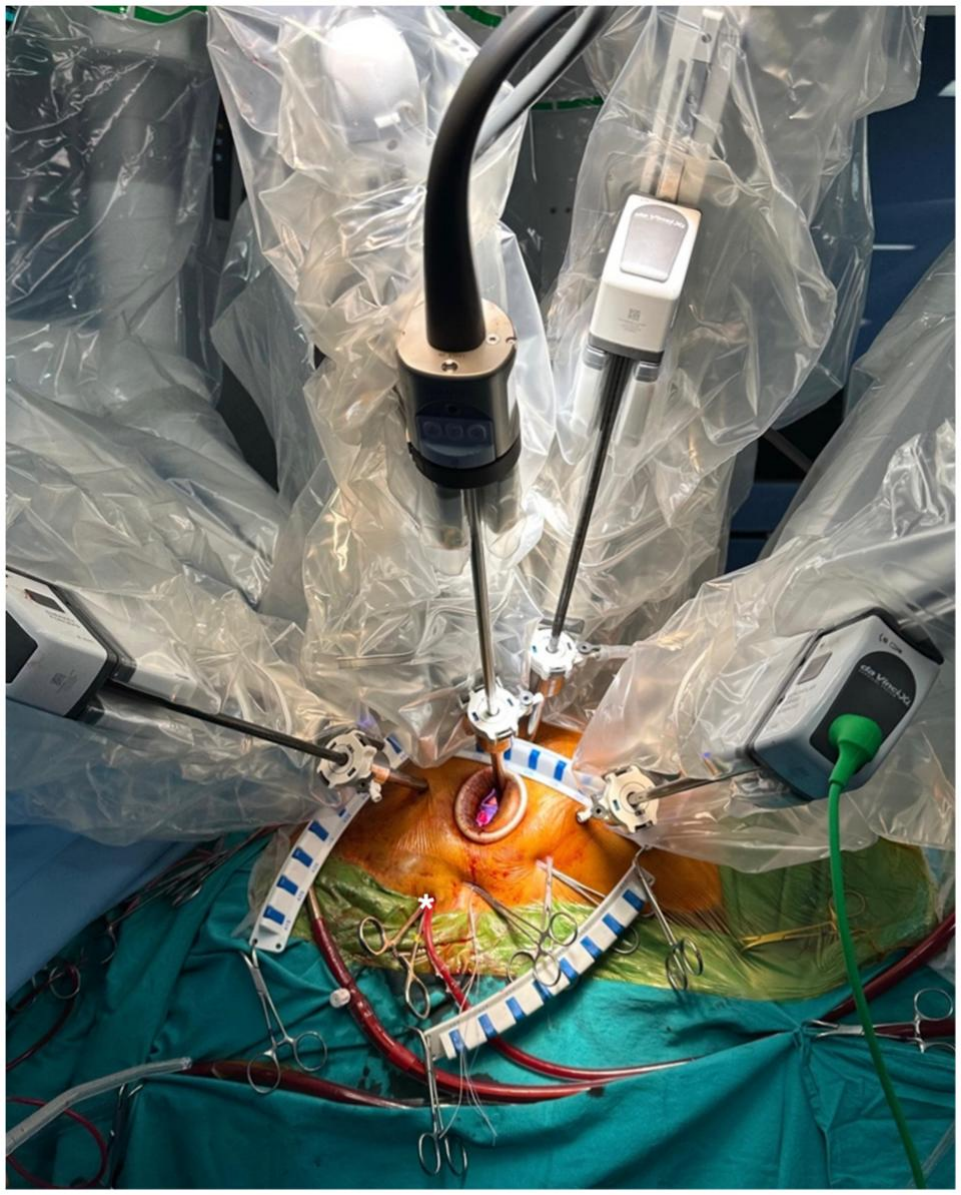
The Robotic Mitral Setup and Placement of the Left Atrial Vent Through a Stab Incision at the Second Intercostal Space Through the Anterior Axillary Line With the Chitwood Clamp Marked with an (*).

After left atriotomy, a small incision was made to the pericardium above the phrenic nerve, and a transpericardial left atrial vent was placed through this incision (**Video 1**). Mitral repair with ring implantation and artificial chorda placement can be seen in this surgical video. Generally, the vent is placed over the pericardiotomy and thus not placed in a fixed position. This technique helps with keeping the vent in correct position, achieving a bloodless working field and avoiding the obstruction of exposure during the procedure. The penetration of the vent when sent through the pericardium is much better when compared to suture fixation or rigid malleable vents as the vent can be positioned closer to the left atrium posterior wall and thus does not conflict with or limit the surgical site. As shown in the surgical setup in **Figure 1**, the vent is not introduced through the mini-thoracotomy but through the cross-clamp puncture site, thus it is not possible to introduce a malleable, rigid vent through this site. This is quite important as we perform a mini-thoracotomy and use only a XXS soft tissue retractor, and an extra vent passing through this site limits the surgical working space. In some cases, like robotic atrial septal defect repair, we do not perform a thoracotomy and only operate through the robotic ports, thus sending a rigid malleable vent is also not possible in these cases. Placing the vent through the cross-clamp site also prevents collision with other surgical tools and displacement of the vent.

As we worked in a limited space during the minimally invasive and robotic setup, this technique is a good solution for venting that does not limit our field of vision. The same technique was also used to place a left ventricular vent through a second incision to the pericardium, especially in robotic mitral surgery cases done under fibrillation. Two vents, one left atrial and the other left ventricular, sent through the pericardium were used throughout the case, where redo robotic mitral repair was performed (**Video 2**). Important technical points to consider are to visualize the phrenic nerve and to do the pericardial incision right under the pericardiotomy with a distance of at least 2 cm between the nerve trace and the vents to avoid any injury to the nerve. Positioning the vents as posteriorly as possible helps achieve a better field of vision. Avoiding cardiac distension is of utmost during this procedure. Especially in cases done under fibrillation, left ventricular distention may result in subendocardial ischaemia. This technique helps with achieving the correct position for effective venting and also for performing a comfortable procedure with good exposure. It also helps reduce the interference of the vents with other tools introduced into the field through the working port and thus avoids the dislodgement of the vents.

This practical and easy-to-implement technique may be beneficial to avoid important complications such as air embolisms and left ventricular distension resulting in subendocardial ischaemia during robotic mitral valve procedures. The fixation of the sucking vents through pericardial incisions helps the surgeon achieve a better exposure and eliminates the need to reposition the vents multiple times during the procedure.

## Data Availability

Data of the study will be made available upon request. No material was reproduced from other sources.
